# Assessment of VP Shunt Valve Settings Using Reconstructions from Non-Contrast Head CT: A Comparative Study with Conventional Radiographs

**DOI:** 10.1007/s00062-025-01606-4

**Published:** 2026-01-14

**Authors:** Raya Juliane Ocker-Serger, Hanna Styczen, Yan Li, Maximilian Schuessler, Marcel Opitz, Sebastian Zensen, Berk Yildirim, Laura Klüner, Benjamin Schroeer, Johannes Haubold, Thiemo Dinger, Philipp Dammann, Ulrich Sure, Michael Forsting, Cornelius Deuschl, Denise Bos

**Affiliations:** 1 Institute of Diagnostic and Interventional Radiology and Neuroradiology, Essen University Hospital, Essen, Germany; 2 Faculty of medicine, University of Duisburg-Essen, Essen, Germany; 3 Institute of Neurosurgery and Spinal surgery, Essen University Hospital, Essen, Germany; 4 Institute of Diagnostic and Interventional Radiology, University Hospital of Zurich, Zurich, Switzerland; 5 Department of Diagnostic and Interventional Radiology, University Hospital Heidelberg, Heidelberg, Germany

**Keywords:** Radiation Exposure, Cerebrospinal Fluid Shunts, Hydrocephalus, Image Processing, Maximum intensity projection, Computed tomography

## Abstract

**Purpose:**

The aim of the study was to investigate whether the ventriculoperitoneal (VP) shunt valve setting can be reliably assessed using maximum intensity projection (MIP) reconstructions from non-contrast, full-dose head CT scans, and how this method performs in comparison to conventional lateral skull radiographs.

**Methods:**

This retrospective study included 41 adult patients (mean age 59 ± 25 years) with Codman Certas programmable VP shunt valves who underwent lateral skull X‑ray and a same-day, non-contrast head CT scan between January and July 2024. From the CT data, MIP reconstructions of the valve region were generated. Three neuroradiologists, blinded to each other’s assessments, independently rated valve settings and image quality using a 5-point Likert scale. Mean reconstruction time was recorded. Radiation dose data were extracted from institutional dose-monitoring software.

**Results:**

Valve settings were identifiable in all 44 CT/X-ray image pairs, with 95% agreement between MIP and X-ray readings. MIP reconstructions were successfully generated (median CTDI_vol_ 35.34 mGy (30.42; 40.32); mean reconstruction time 70 s). Image quality was rated lower for MIP (median 2 [IQR 2]) than for X‑ray (median 4 [IQR 1]; *p* < 0.001). In 45% of cases scanned with photon-counting CT, MIP quality was significantly higher (median 3 vs. 1; *p* < 0.001). Inter-reader reliability was good for MIPs (ICC = 0.82) and excellent for X‑rays (ICC = 0.97).

**Conclusion:**

MIP reconstructions from non-contrast head CT allow reliable VP shunt valve setting assessment and may reduce the need for additional radiographs, especially when advanced CT systems are used.

## Introduction

A ventriculoperitoneal (VP) shunt is used to treat hydrocephalus by draining cerebrospinal fluid from the brain’s ventricular system into the abdominal cavity [1]. Due to the high failure rate of VP shunts [2], patients often undergo multiple CT and X-ray scans throughout their lives to monitor the shunt’s position, material damage and the VP shunt valve setting. The Codman Certas Programmable Valve allows for regulation of eight adjustable pressure settings, allowing non-invasive modification of the opening pressure. Settings 1 to 7 cover a range from 25 to 215 mmH_2_O, while the eighth setting, known as “Virtual Off”, provides an opening pressure exceeding 400 mmH_2_O, effectively minimizing cerebrospinal fluid flow [3–5]. A precise and reliable measurement of the shunt valve setting is essential to ensure proper functioning, as incorrect adjustment can lead to severe complications, such as insufficient or excessive drainage of cerebrospinal fluid [6]. X‑ray is considered as the standard for assessing VP shunt settings [7]. Repeated radiation exposure increases the long-term risks associated with ionizing radiation [8]. As patients with hydrocephalus frequently require lifelong monitoring and possible shunt revisions, minimizing radiation exposure becomes increasingly important. In addition to health concerns, frequent imaging also imposes logistical and financial burdens on healthcare systems. Therefore, identifying alternative imaging strategies that maintain diagnostic accuracy while reducing resource utilization is a clinically relevant goal. In clinical practice, however, cranial CT is the gold standard in emergency diagnostics for suspected VP shunt dysfunction, as it allows rapid assessment of ventricular size, shunt position and associated complications. In such cases, reusing the already available CT data for valve assessment via MIP reconstructions offers a pragmatic approach. This would avoid the need for an additional X‑ray examination, thereby saving unnecessary radiation exposure and streamlining workflow without compromising diagnostic accuracy. Although a few studies have explored the use of CT imaging to assess programmable shunt valve settings [9–11], they are limited by methodological constraints such as small sample sizes, non-standardized reconstruction techniques, and lack of direct comparison to radiographic reference standards.

Thus, further research is needed to evaluate whether MIP reconstructions from already acquired CT scans can reliably support valve setting assessment and help avoid additional radiographs in routine clinical scenarios. Therefore, the aim of this retrospective study was to investigate whether the VP shunt valve setting can be reliably assessed from MIP reconstructions of full-dose, non-contrast head CT scans and how this method performs in comparison to conventional radiographs.

## Methods

### Patient Cohort

**Fig. 1:**
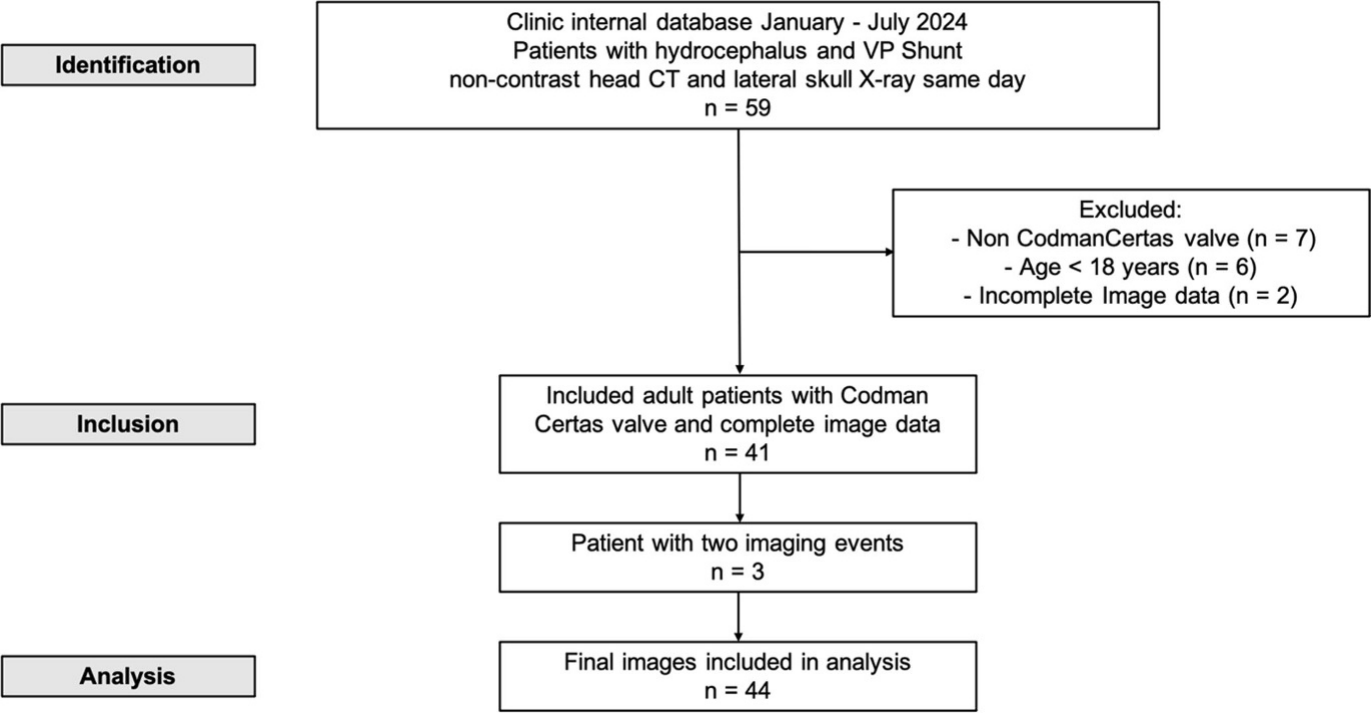
Flowchart of patient selection

We retrospectively identified 59 patients with implanted VP shunt system who had undergone both non-contrast head CT and lateral skull X‑ray in the clinical routine between January and July 2024 by searching our clinic internal database tool (Essen, Germany). After excluding patients with non-Codman Certas valves (*n* = 7), individuals under 18 years of age (*n* = 6), and those with incomplete imaging data (*n* = 2), a final cohort of 41 adult patients remained. All included patients with Codman Certas® Plus Programmable Valve (Integra LifeSciences, Princeton, New Jersey, USA) received both imaging modalities on the same day and under the same protocol (Fig. 1). Three patients underwent imaging on two separate occasions, resulting in a total of 44 matched CT/X-ray image pairs. The cohort was 43% male and 57% female, with a mean age of 59 ± 25 years (range 18–87).

### Imaging Acquisition and MIP Reconstruction

All X‑ray and CT scans were conducted using standard clinical protocols for diagnostic purposes. CT scans were acquired using multi-slice scanners with submillimeter resolution (slice thickness 0.6–1.0 mm), all from Siemens Healthineers (Erlangen, Germany), Table 1. In 21 of 44 imaging cases (48%), scans were performed on a photon-counting CT system (NAEOTOM Alpha, Siemens Healthineers), while the remaining 23 were acquired using conventional energy-integrating detector CT scanners. Subgroup analysis was performed accordingly. Skull X‑rays were acquired in lateral projection using standard digital radiography systems (Ysio, Siemens Healthineers). MIP reconstructions were created from the CT dataset by using Syngo.via workflow (Siemens Healthineers, Erlangen, Germany). Windowing parameters were adjusted for each case to best display the shunt valve. The mean reconstruction time per case was recorded. Reconstructions were performed by a radiologist experienced in MIP post-processing. To read the shunt setting, the image file must be mirrored at the end. Furthermore, the MIP reconstructions were rotated to match the orientation of the manufacturer’s reference scale used for valve setting identification. The time required for reconstruction was measured.

**Table 1:** Patient characteristics and CT examinations.

Characteristics		Number of patients	[%]
Patients	Total	41	100
	Female	23	56
	Male	18	44
Age distribution	20–30	2	5
	30–40	7	17
	40–50	7	17
	50–60	6	15
	60–70	12	29
	70–80	3	7
	≥80	4	10
Number of patients with	One examination	38	93
	Two examinations	3	7
CT Scanner (all Siemens)	NAEOTOM Alpha	20	45
	SOMATOM Definition AS+	2	5
	SOMATOM Definition Edge	18	41
	SOMATOM Force	3	7
	SOMATOM X.ceed	1	2
CT Protocol	CCT_nativ	41	9
	Stroke	3	7
Shunt valve type	Codman Certas	44	100

### Image Evaluation

Three board-certified neuroradiologists independently reviewed the CT MIPs and corresponding lateral skull X‑rays. For each image set, readers recorded: First, the valve setting based on comparison with manufacturer reference images, and second, a subjective image quality score using a 5-point Likert scale (1 = non diagnostic to 5 = excellent).

### Radiation Dose Analysis

CT Radiation dose indices were extracted from Radimetrics^TM^, a commercial dose monitoring software (Bayer HealthCare LLC, Whippany, NJ, USA). For CT, we recorded CTDI_vol_, dose-length product (DLP), and effective dose (ED) and for X‑rays, the dose-area product (DAP). The ED was calculated by multiplying the DLP by an age-specific conversion factor (k = 0.002 mSv·mGy⁻^1^·cm⁻^1^), as proposed by Romanyukha et al. [12]. This method is based on Monte Carlo simulations using anthropomorphic phantoms and allows for more accurate dose estimation across varying patient sizes and anatomical regions. For X‑ray examinations, the effective dose was calculated by applying a consistent conversion factor (k = 0.006 mSv·Gy⁻^1^·cm^2^⁻^1^) implemented in the dose monitoring software PCXMC 2.0.

### Statistical Analysis

The descriptive statistics and statistical analysis were performed with the Statistical Package for Social Sciences v. 26.0 (SPSS Inc., New York, USA). To determine normal distribution Kolmogorov-Smirnov test was applied. Normally distributed data are reported as mean ± standard deviation (SD), non-normally distributed data as median and interquartile range (IQR). The radiation dose of CT (CTDI_vol_, DLP, and ED) and X-ray (DAP) was determined. A *p*-value lower than *p* < 0.05 was considered statistically significant. Inter-reader agreement was calculated using intraclass correlation coefficients (ICC) based on a two-way random effects model with absolute agreement and single measures (ICC (2,1)).

## Results

**Fig. 2:**
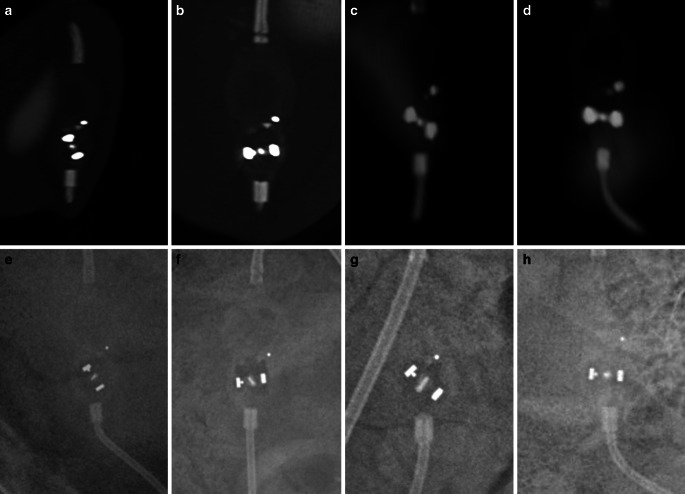
Comparison of Codman Certas VP shunt valves in CT MIP reconstructions using photon-counting CT (**a**, **b**) and third-generation CT (**c**, **d**), compared with lateral skull radiographs. The valves are shown at pressure settings 5, 4, 5, and 4 (from left to right)

A total of 41 adult patients (mean age 59 ± 25 years, range 18–87) with Codman Certas programmable VP shunt valves were included after applying predefined criteria as shown in Fig. 1. Three patients underwent repeat imaging, resulting in 44 CT/X-ray pairs for comparative analysis. In 45% of cases, imaging was performed on a photon-counting CT scanner. From each CT scan, MIP reconstructions of the valve region were successfully generated. Patient characteristics and CT examinations as shown in Table 1. The median CTDI_vol_ was 35.34 mGy (30.42; 40.32), and the DAP of the corresponding lateral skull X‑rays averaged 0.119 mGy·cm^2^ (0.582; 0.349). The estimated ED of the CT scan was 1.06 mSv (1.00; 1.20; Table 2). Valve settings were identifiable in all MIP reconstructions, with 95% agreement compared to X‑ray-based readings. The mean reconstruction time was 70 s (range 38–300), representing a quick and feasible post-processing step. Subjective image quality was rated lower in MIPs (median 2, IQR 2) than in X‑rays (median 4, IQR 1), *p* < 0.001. Qualitatively, MIP reconstructions were frequently affected by hardening artifacts, leading to local overexposure and blurred valve contours, whereas X‑rays provided sharper delineation of the structural details with less visual distortion as shown in Fig. 2. A subgroup of 21 patients examined using a photon-counting CT system showed significantly higher subjective image quality ratings across all three raters (median 3, IQR 1) compared to those scanned with conventional CT systems (median 1, IQR 1; *p* < 0.001). However, diagnostic interpretability of the valve setting was consistently maintained. Inter-reader reliability was good for MIPs (ICC = 0.82) and excellent for X‑rays (ICC = 0.97), indicating high reproducibility across modalities and readers.

**Table 2:** Dose data.

Modality	Dose Parameter	Median (IQR)
CT	CTDI_vol_ (mGy)	35.3 (30.42; 40.32)
	DLP (mGy · cm)	528 (473.07; 621.02)
	ED ICRP 103 (mSv)	1.06 (1.00; 1.20)
	mAs	199 (63; 246.54)
	kV	100 (100; 120)
X‑ray	DAP (Gy · cm^2^)	0.119 (0.582; 0.349)
	ED (mSv)	0.0007 (0.0003; 0.0021)
	mAs	10.5 (6.4; 18.95)
	kV	65.9 (65.90; 69.80)

## Discussion

We investigated whether VP shunt valve settings can be reliably assessed using MIP reconstructions derived from non-contrast head CT scans. The results demonstrated a high diagnostic accuracy between MIP- and radiograph-based assessments and good inter-reader reliability, supporting the reproducibility of this approach.

Although lateral skull X‑ray remains the gold standard due to its superior reproducibility [3], our findings suggest that MIP reconstructions can serve as a reliable adjunct when a head CT has already been performed for other clinical indications, such as evaluating ventricular size, shunt positioning, or neurological symptoms [9, 10]. This reflects common clinical practice and highlights the potential to avoid additional radiographic examinations through secondary use of CT data.

In contrast to the CT scan, the additional radiation dose from a single lateral skull radiograph is low and approximates 0.002 mSv [13]. Lens exposure is typically minimal, especially if the orbits are excluded from the primary beam. Head CTs typically contribute 1.6 mSv, and in rare cases abdominal CTs used to assess the distal shunt catheter may exceed 7.9 mSv [14]. While radiation dose is not the primary justification for using MIP reconstructions, the ability to avoid redundant imaging may contribute to cumulative dose reduction and improve resource utilization.

MIP reconstruction was technically feasible in all cases and required a mean of 70 s per dataset, which represents a relatively short processing time compared to the time and resources needed for a separate X‑ray. This pragmatic approach can reduce total time for image acquisition and reporting, streamline workflow and help reduce the costs for an additional X‑ray [15]. This can be especially beneficial in busy healthcare settings. Given the frequency of diagnostic imaging for VP shunt malfunction [16], strategies that reduce redundant examinations without compromising diagnostic accuracy are of clinical relevance.

PCCT scanners, such as the NAEOTOM Alpha (Siemens Healthineers), yielded significantly higher image quality ratings for MIP reconstructions compared to conventional CT systems. This improvement was consistent across all three readers and supports the assumption that PCCT enhances valve visualization and may facilitate more reliable MIP-based assessment. As PCCT technology becomes increasingly available in clinical practice, it could further support the implementation of CT-based valve evaluation, particularly to avoid additional radiographic imaging.

While MIP reconstructions demonstrate clear potential, several limitations must be acknowledged. Image quality was rated lower for MIPs than for radiographs, particularly in cases scanned using conventional CT systems. Although the shunt valve setting could be reliably assessed in all cases, reduced image quality may cause uncertainty—especially for radiologists with less neuroradiological experience.

Since incorrect interpretation or documentation of valve settings can have significant clinical and even legal consequences (e.g. subdural haematoma due to overdrainage caused by incorrect low-pressure settings), it should be emphasised that, despite the high degree of consistency in our cohort, the sample size was limited.

Moreover, the diagnostic performance of MIP reconstructions relies on the availability of high-quality CT data. In situations when image quality is compromised due to a restless patient or technical issues the utility of MIP reconstructions may be limited and an additional X‑ray has to be added. It is also important to note that the reconstruction time varied between 38 and 300 s in our study, which may affect the workflow in clinical settings, particularly in scenarios where rapid assessments are required. In our study, all reconstructions were performed by a radiologist, and some initial training may be required to integrate this technique into clinical routine.

Our study included only adult patients from one medical center and one type of programmable shunt valve (Codman Certas). Furthermore, all CT examinations were performed on scanners from a single vendor, which may limit transferability to other systems. Therefore, generalizability to pediatric populations, who are more vulnerable to cumulative radiation effects due to repeated imaging [17] and often undergo low-dose protocols [18], as well as to other valve models, may be limited. In addition, low-dose protocols were not evaluated in our study, neither in adults nor in children.

Future studies should confirm these findings in larger, multicenter cohorts and across various valve systems. It might also be interesting to define a threshold value for image quality—for example, a limit value on a Likert scale—above which the valve pressure can be determined with high reliability and below which a conventional X‑ray should still be taken. This concept could serve as a guide for clinical decision-making and form the basis for a subsequent prospective project. The potential of photon-counting CT for high-resolution visualization should be further evaluated. AI-based tools for automated valve identification and setting classification may further reduce inter-reader variability and improve clinical workflow [19]. This could contribute to long-term dose reduction and improve imaging efficiency [20]. However, given the relatively low dose of a single skull radiograph, the primary justification for MIP use should lie in workflow optimization rather than dose reduction alone.

## Conclusion

MIP reconstructions from full-dose, non-contrast head CT enable reliable assessment of VP shunt valve settings. As a secondary use of existing CT data, this approach can reduce additional radiographs and streamline clinical workflow. With the growing availability of photon-counting CT and AI-assisted analysis, its clinical value is likely to increase further.

## Abbreviations

VP: Ventriculoperitoneal
MIP: Maximum intensity projection
CTDI_vol_: Volumetric CT dose index
DAP: Dose area product
PCCT: Photon-counting CT
